# Evaluation of SARS-CoV-2 genome detection by real-time PCR assay using pooled specimens

**DOI:** 10.2217/fvl-2021-0014

**Published:** 2022-05-09

**Authors:** Forough Tavakoli, Jila Yavarian, Nazanin Zahra Shafiei Jandaghi, Kaveh Sadeghi, Nastaran Ghavami, Vahid Salimi, Talat Mokhtari-Azad

**Affiliations:** ^1^Virology Department, School of Public Health, Tehran University of Medical Sciences, Tehran, Iran; ^2^Department of Bacteriology and Virology, Faculty of Medicine, Isfahan University of Medical Science, Isfahan, Iran

**Keywords:** genome detection, pooled samples, pooling method, RT-qPCR, SARS-CoV-2

## Abstract

**Objective:** To evaluate SARS-CoV-2 genome detection using pooled samples by RT-qPCR assay, compared to individual samples. **Method:** At first all samples were tested individually using two commercial methods targeting *ORF1ab*, *NP* and *E* genes. Then, four experimental groups of samples were pooled and evaluated using the same detection methods. **Findings:** Compared to the individual sample testing, the sample pooling conserved the sensitivity of the detection in all groups of pooled samples when the Ct value in single test was lower than 33. **Conclusion:** Specimen pooling may fail to detect positive samples with high Ct values. However, in scarcity of reagents or in population surveys, it could be considered as an alternative method in low prevalence settings.

## Introduction

Since SARS-CoV-2's first emergence in Wuhan (China), late December 2019, it caused a large pandemic [1,2]. The first detection of SARS-CoV-2 in Qom, Iran, was officially announced on February 19, 2020 [3]. As of May 4, 2022, more than 512,607,587 cases of COVID-19 and 6,243,038 deaths have been reported globally. In Iran from 3 January 2020 to 4 May 2022, about 7,223,576 confirmed cases and 141,131 deaths were reported [4]. SARS-CoV-2 has now become a major threat to public health around the world [5].

The variable incubation period of COVID-19 [6], the role of asymptomatic carriers in its transmission [7] and the rapid spread of SARS-CoV-2 [8–10] highlight the prominence of a practical surveillance program to improve COVID-19 management in the society. Therefore, COVID-19 outbreak surveillance needs analysis of a mass population for an accurate diagnosis or to establish that a population is free of COVID-19 disease [11,12]. A routine laboratory method for SARS-CoV-2 infection detection both in symptomatic and asymptomatic patients is real-time PCR [13]. However, early in the pandemic, the world experienced a shortage of diagnostic kits for SARS-CoV-2 [5]. Therefore, instead of testing each sample individually, a few laboratories introduced the pooling of several specimens for COVID-19 [5,11,13–15]. This was specially performed in asymptomatic carriers with low infection rates in the screening programs. For interpretation, if the result of real-time PCR for a pooled sample was negative, all samples were considered negative. If the result of any pooled sample was positive, each sample should be retested individually to find a positive sample or samples. The group testing has previously been employed for the detection of the human immunodeficiency virus, hepatitis B and C viruses and influenza virus [16,17]. Nevertheless, as the positive samples might be diluted in a mixture of negative samples, this could result in reducing the test's sensitivity. Thus, group testing should be evaluated for each real time method before it is used as a routine diagnostic test for SARS-CoV-2. As this evaluation has not been performed in Iran or even other countries in Middle East, the present study was performed to assess the pooling method's sensitivity to detect SARS-CoV-2 genome using two common and available approaches of real-time PCR assay.

## Method

The throat swabs in viral transport medium were sent routinely via Ministry of Health and Education to the Iran National Influenza Center (NIC) located at Tehran University of Medical Sciences, for SARS-CoV-2 testing under cold chain transport. A one-step real-time RT-PCR for SARS-CoV-2 has been routinely performed on all specimens. To evaluate the usefulness of pooling samples of SARS-CoV-2 genome detection by real-time PCR, from previously tested specimens for routine diagnosis, 15 positive specimens with CT value of 16–40 and 29 negative samples were randomly chosen. Then, 60 experimental pools were created using 200 microliters of one SARS-CoV-2-positive throat specimen mixed with four batch sizes comprising of 4, 9, 19 and 29 negative samples (100 microliters each; Table 1). The size of each batch was selected based on previous studies [5,11,13].

**Table 1.** Detection of SARS CoV-2 RNA by RT-PCR in pooled throat specimens from patients with COVID-19/*ORF1ab* gene, NP gene, 200 μL non-pooled positive *ORF1ab/NP* gene sample was added to each of negative pool sizes of 4, 9, 19, and 29.

Sample no.	Non-pooled (Ct) *ORF1ab* gene	Positive throat specimen pooled with 4 negative throat specimens (Ct) *ORF1ab*	Positive throat specimen pooled with 9 negative throat specimens (Ct) *ORF1ab*	Positive throat specimen pooled with 19 negative throat specimens (Ct) *ORF1ab*	Positive throat specimen pooled with 29 negative throat specimens (Ct) *ORF1ab*	Non-pooled (Ct) *NP* gene	Positive throat specimen pooled with 4 negative throat specimens (Ct) *NP*	Positive throat specimen pooled with 9 negative throat specimens (Ct) *NP*	Positive throat specimen pooled with 19 negative throat specimens (Ct) *NP*	Positive throat specimen pooled with 29 negative throat specimens (Ct) *NP*
1	16	17	19	22	28	16	20	22	30	28
2	24	25	27	29	28	24	29	31	31	31
3	25	27	29	29	30	25	31	33	33	33
4	26	29	31	32	33	26	33	34	35	35
5	28	30	31	33	33	28	31	33	34	34
6	29	30	31	32	33	29	32	33	35	36
7	30	31	31	33	32	26	32	33	35	34
8	32	34	35	35	35	34	37	36	39	39
9	33	ND	ND	ND	ND	35	37	37	37	ND
10	34	ND	ND	ND	ND	36	ND	ND	ND	ND
11	35	ND	ND	ND	ND	35	37	38	37	ND
12	36	ND	ND	ND	ND	37	ND	ND	ND	ND
13	38	ND	ND	ND	ND	39	ND	ND	ND	ND
14	39	ND	ND	ND	33	40	ND	ND	ND	38
15	40	ND	ND	ND	ND	40	ND	ND	ND	ND

A volume of 200 μL/specimen was used for preparing pools.

ND: Not determined.

For both individual and pooled samples, 200 μl of specimens were subjected to viral RNA extraction using the High Pure Viral Nucleic Acid Kit (Roche, Mannheim, Germany), according to the manufacturer's instruction. Extractions were stored at -80°C until the next steps. A one-step real-time RT-PCR was performed using two methods. The first method targeted the *ORF1ab* and *NP* genes using Sansure kit (Novel Coronavirus [2019-nCOV] Nucleic Acid Diagnostic Kit, Sansure Biotech Inc., Changsha, China). The second method involved targeting primers and probes for *E* gene (LightMix ^®^ Sarbeco V *E* gene plus EAV control kit, MOLBIOL, Berlin, Germany) using Invitrogen master mix and enzyme (SuperScript III Platinum One-Step qRT-PCR Kit). The assays were conducted on a StepOnePlus ^™^ Real-Time PCR Systems (Thermo Fisher Scientific, MA, USA) following the manufacturer's instructions. In the next step, *E* gene-positive samples were tested using the RpRd gene (LightMix^®^ Sarbeco V RpRd gene plus EAV control kit, MOLBIOL) recommended by WHO. The cutoff PCR Ct of both methods was ≤40.

## Result

**Figure 1.**
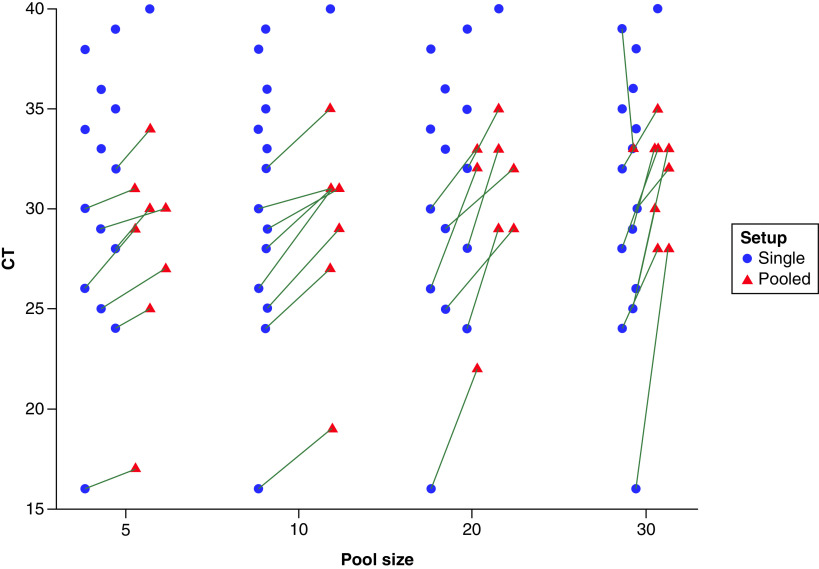
Ct values of single compared with pooled samples. Ct values of positive-pooled samples regarding pool size and corresponding Ct values of single positive samples for the *ORF1ab*-gene assay. Absolute Ct values of positive pools (33 of 60 tested pools) regarding pool size and corresponding Ct values of individual positive samples for the ORF1ab-gene assay. Absolute Ct values were less than or equal to 35 for all pool sizes. Six positive individual samples with Ct values greater than 33 showed non-detectable pools.

**Figure 2.**
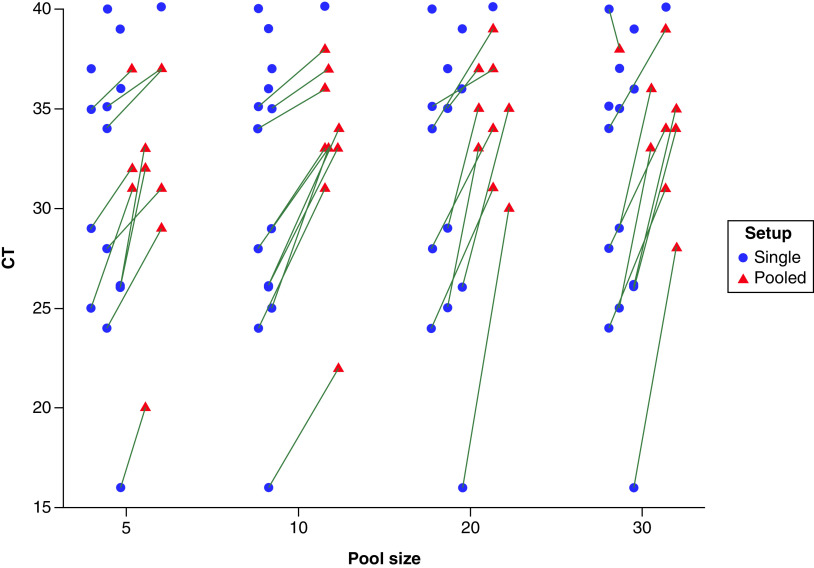
Ct values of single compared with pooled samples. Ct values of positive-pooled samples regarding pool size and corresponding Ct values of single positive samples for the *NP*-gene assay. Absolute Ct values of positive pools (39 of 60 tested pools) regarding pool size and corresponding Ct values of individual positive samples for the *NP*-gene assay. Absolute Ct values were less than or equal to 39 for all pool sizes. Three positive individual samples with Ct values greater than 36 showed non-detectable pools.

**Figure 3.**
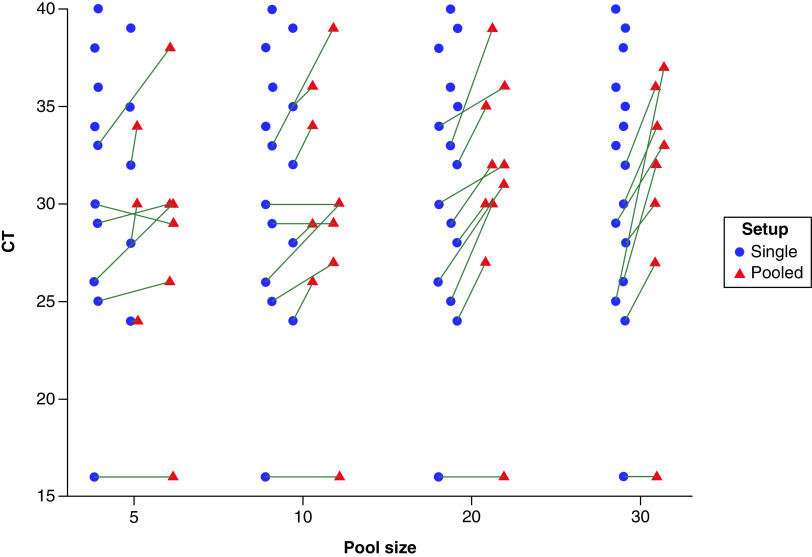
Ct values of single compared with pooled samples. Ct values of positive-pooled samples regarding pool size and corresponding Ct values of single positive samples for the *E*-gene assay. Absolute Ct values of positive pools (37 of 60 tested pools) regarding pool size and corresponding Ct values of individual positive samples for the *E*-gene assay. Absolute Ct values were less than or equal 39 for all pool sizes. Five positive individual samples with Ct values greater than 33 showed non-detectable pools (except 2 pools including a 20 pool size with individual Ct-positive sample of 34 and a 5 pool size with individual Ct-positive sample of 35).

As shown in Figure 1 & Table 1, by using a commercial kit targeting the *ORF1ab* gene, SARS-CoV-2 was detected providing non-pooled positive samples displaying RT-PCR Ct <33 in all groups of 5, 10, 20 and 30 samples. The negative pooled samples did not have any CT and were not seen in the figure. *NP*-positive samples were detected as long non-pooled positive samples displaying RT-PCR Ct ≤35 in groups of 5, 10 and 20 samples and Ct ≤34 in the group of 30 (Figure 2 & Table 1). Negative pooled tests do not have a CT and were not seen on the Figure. Using LightMix ^®^ Sarbeco V *E* gene plus EAV control kit, the *E* gene was detected in groups of 5, 10 and 20 samples, as long undiluted positive samples displaying RT-PCR Ct ≤33. In the sample groups of 30, positive samples were detected which belonged to the non-pooled positive samples up to cycling threshold 32 (Figure 3 & Table 2). As expected, the Ct values were higher in pooled samples.

**Table 2.** Detection of SARS CoV-2 RNA by RT-PCR in pooled throat specimens from patients with COVID-19/*E* gene, 200 μL non-pooled positive *E* gene sample was added to each of negative pool sizes of 4, 9, 19 and 29.

Sample no.	Non-pooled (Ct) *ORF1ab* gene	Positive *NP* specimen pooled with 4 negative NP specimens (Ct)	Positive *NP* specimen pooled with 9 negative *NP* specimens (Ct)	Positive *NP* specimen pooled with 19 negative *NP* specimens (Ct)	Positive *NP* specimen pooled with 29 negative *NP* specimens (Ct)
1	16	16	16	16	16
2	24	24	26	27	27
3	25	26	27	30	37
4	26	30	30	31	32
5	28	30	29	30	30
6	29	30	29	32	33
7	30	29	30	32	34
8	32	34	34	35	36
9	33	38	39	39	ND
10	34	ND	ND	36	ND
11	35	ND	36	ND	ND
12	36	ND	ND	ND	ND
13	38	ND	ND	ND	ND
14	39	ND	ND	ND	ND
15	40	ND	ND	ND	ND

A volume of 200 μL/specimen was used for preparing pools.

ND: Not determined.

The samples that tested positive for the *E* gene confirmed by *RpRd* gene (LightMix ^®^Sarbeco V RpRd gene plus EAV control kit, MOLBIOL) recommended by WHO. The result is shown in Table 3.

**Table 3.** Detection of SARS CoV-2 RNA by RT-PCR in pooled throat specimens from patients with COVID-19/(*RpRd*) gene.

Sample no.	Non-pooled (Ct) *ORF1ab* gene	Positive *NP* specimen pooled with 4 negative *NP* specimens (Ct)	Positive *NP* specimen pooled with 9 negative *NP* specimens (Ct)	Positive *NP* specimen pooled with 19 negative *NP* specimens (Ct)	Positive *NP* specimen pooled with 29 negative *NP* specimens (Ct)
1	16	18	19	22	21
2	24	28.5	32	31	32
3	25	31	32	33	34
4	26	33	34	34	35
5	28	33	34	34	34
6	29	34	34	35	35
7	30	33	35	ND	ND
8	32	38	37	35	35
9	33	ND	ND	ND	ND
10	34	ND	ND	ND	ND
11	35	ND	ND	ND	ND

Samples that tested positive for the *E* gene were confirmed by the *RpRd* gene.

ND: Not determined.

## Discussion

This research aimed to evaluate the utility of pooling specimens for SARS-CoV-2 genome detection by the real-time PCR method. In this study, positive SARS-CoV-2 clinical samples with different Ct values were used to assess the viral load effect of individual specimens. This approach evaluated the test's positivity using pooled samples in the real-time PCR method. Besides, to find the best batch size of pooling, four groups with different sizes were analyzed. Our results depicted that the detection of a positive clinical sample was independent of the pool size (5, 10, 20 and 30). That is, provided the Ct value of *Orf1ab* real-time RT-PCR for the original individual specimen was <33 in pooled samples, one positive clinical sample could be detected. Positive samples with a low viral load (Ct value ≥33) in pooled samples were not detected (Figure 1). It should be recalled that, the infectivity risk of individuals with low viral load is expected to be low [14,18–22]. Therefore, in the COVID-19 survey, the consequences of missing such types of samples on the health system might be negligible. However, serious concerns remain about individuals who are at higher risk of severe COVID-19 infection or hospitalized patients.

This study was the first research using pooled samples for COVID-19 diagnosis with common and available real-time PCR methods in Iran. It was performed during May 2021 when the infection prevalence was 0.15%. The results showed both methods preserved the sensitivity of the tests to detect a positive sample except when the Ct value of the non-pooled positive sample was higher than 33 (Figures 1–2). This finding is consistent with other investigations suggesting that the pooling strategy had enough diagnostic accuracy until positive samples in pools had a high-to-intermediate viral load [14,23,24]. The results of confirmation by *RpRd* gene in Table 3 show slightly different results to *E* gene in Table 2, with generally higher Ct values in Table 2 compared to Table 1. Although this slight difference may be explained by targeting 2 different SARS-CoV-2 genes, but may be due to the presence of subgenomic mRNA also being detected with these primer/probes targeting the *E* gene giving lower Ct values compared to the *ORF1ab*-targeting primers. Herein, Ct variations between original samples and pooled samples were in the range of 2.0 to 4.0 units in the group of 5 samples and 3.0 to 5.0 in the group of 10 samples. It was hypothesized that, if the Ct difference is less than 3 in any of the studied batch size, a positive sample with the Ct up to 35 might be detectable when pooled with negative samples. Also, if the Ct difference is greater than 3, the likelihood of a false-negative result would increase. In this study, a range of 5 to 30 samples per pool showed similar real-time RT-PCR results, consistent with previous reports performed on the similar sample sizes per pool [13,14,23]. To find the optimal sample pool size, predictive algorithmic principles depending on the parameters such as the prevalence rate, the false positivity and the false negativity rate of the implemented tests were used [11,25]. It had been previously reported that the optimal infection rate pool size for 0.1 %, 1% and 10% is about 32, 11 and 4, respectively [25]. For an infection rate above the range of 15% to 29% the optimal pool size is 3 [25]. Though, another report suggested that a prevalence of around 20% may enable substantial savings in a group of 10 samples [11]. Pooled testing was not valuable for infection levels of 30% and higher [25]. Population screening is a crucial and recommended strategy for COVID-19 control [11,12]. To follow this strategy, a huge amount of laboratory materials is needed. Sample pooling minimizes the use of reagents, increases the testing capacity and saves time. The lineage B2 was more prevalent in the study population. Different SARS-CoV-2 lineages could potentially affect the sensitivity of PCR-based detection methods. Simultaneous detection of more than one fragment of the SARS-CoV-2 genome could overcome the false-negative challenge in both individual and pooled samples tests [26,27]. This method has disadvantages, as well. On one hand, the samples with high PCR Ct in single tests (Ct ≥30) have shown lower Ct in the pooling method [5]. On the other hand, the risk of false-negative results in samples containing low viral load has been reported [14,23,28]. The highly sensitive assays may enhance accuracy detection. It should be emphasized that sample pooling for SARS-CoV-2 detection is just applicable in low prevalence areas or low-risk populations [23,29–31]. However, technical limitations including personnel error in pipetting during pooling or PCR may influence the result. Also, the sample pooling may increase the possibility of contamination or reaction inhibitors in the swabs. Moreover, we probably cannot make inferences about optimal pool size based on this study because this is more dependent on prevalence. Another limitation of this study is the lack of technical or biological replicates to show accurate differences in cycle threshold between single and pooled specimens. Perhaps it may be better to validate the approach using 15 positive throat specimens before 60 experimental pools for purposes of better flow, in future directions for this study.

## Conclusion

Collectively, our results depict that specimen pooling using common and available RT-PCR testing in Iran and most of the countries in Middle East, might result in missing persons with a mild infection. When there is a shortage of resources, this could be a challenge for persons with a high vulnerability to acute COVID-19 infection or hospitalized patients.

**Summary points**

- Since world experiences a shortage of diagnostic kits for SARS-CoV-2, pooling of several specimens was specially performed in asymptomatic carriers with low infection rates in the screening programs.
- The detection of a positive clinical sample was independent of the pool size up to 30 samples.
- Provided the Ct value of *Orf1ab* real-time RT-PCR for the original individual specimen was <33 in pooled samples, one positive clinical sample could be detected.
- Specimen pooling using the alternative time RT-PCR testing might result in missing persons with a mild infection that this could be a challenge for persons with a high vulnerability to acute COVID-19 infection or hospitalized patients.
